# Clinical Correlates of Awareness for Balance, Function, and Memory: Evidence for the Modality Specificity of Awareness

**DOI:** 10.1155/2014/674716

**Published:** 2014-01-16

**Authors:** Megan E. O'Connell, Vanina Dal Bello-Haas, Margaret Crossley, Debra Morgan

**Affiliations:** ^1^Department of Psychology, Room 183 Arts Building, 9 Campus Drive, University of Saskatchewan, SK, Canada S7N 5A5; ^2^School of Rehabilitation Science, McMaster University, Hamilton, ON, Canada L8S 1C7; ^3^Canadian Centre for Health and Safety in Agriculture, University of Saskatchewan, SK, Canada S7N 5E5

## Abstract

Awareness in dementia is increasingly recognized not only as multifactorial, but also as domain specific. We demonstrate differential clinical correlates for awareness of daily function, awareness of memory, and the novel exploration of awareness of balance. Awareness of function was higher for participants with mild cognitive impairment (aMCI and non-aMCI) than for those with dementia (due to Alzheimer disease; AD and non-AD), whereas awareness of memory was higher for both non-aMCI and non-AD dementia patients than for those with aMCI or AD. Balance awareness did not differ based on diagnostic subgroup. Awareness of function was associated with instrumental activities of daily living and caregiver burden. In contrast, awareness of balance was associated with fall history, balance confidence, and instrumental activities of daily living. Clinical correlates of awareness of memory depended on diagnostic group: associations held with neuropsychological variables for non-AD dementia, but for patients with AD dementia, depression and instrumental activities of daily living were clinical correlates of memory awareness. Together, these data provide support for the hypothesis that awareness and dementia are not unitary and are, instead, modality specific.

## 1. Introduction

Unawareness, lack of insight, or anosognosia refers to impaired awareness in persons with dementia [1–7]. Awareness is multifactorial and likely modular [4, 8–10], with each domain separable and potentially unique. Most of the literature on awareness in persons with dementia describes the clinical correlates of one awareness domain (reviews by [1, 4, 11]), but the few studies that have contrasted awareness for different domains have found differential patterns of clinical correlates [12–15]. This paper provides further support for the modality specific nature of awareness in dementia by contrasting the clinical correlates for awareness of balance in addition to more commonly measured awareness of day-to-day function and memory.

Awareness quantification remains elusive, and there is no consensus method for measuring awareness (e.g., [4, 9]). Awareness has been measured with clinician ratings [16, 17]; or based on discrepancy between self-report versus clinicians' impression [10] or versus informant report assessed with interview [10, 18] or questionnaires [7, 12, 14, 19–21]; or discrepancy between self-report and objective performance [21, 22], which, depending on the task, measures self-monitoring or metacognitive abilities [3]. Each assessment method has limitations: Clare et al. [21] detail difficulties with patient/informant discrepancies and the assumption that caregiver informants' or clinicians' reports are a better reflection of reality. Moreover, worry, anxiety, defensiveness, denial, or focus on “more important problems” can influence reflection for persons with dementia [9]. Caregivers' reports may be more highly correlated with objective measures of cognition than patient self-reports, suggesting there is value in using patient/informant discrepancies [7]. Nevertheless, caregiver/patient discrepancies may be a better measurement of patients' awareness of function, whereas clinician/patient discrepancies may be a better measure of patients' awareness of cognition [11].

Due, in part, to measurement challenges when assessing awareness, the literature on the clinical correlates of awareness in persons with dementia is contradictory [1]. Most, but not all, of the cumulative data suggest increasing dementia severity is associated with reduced awareness [1, 16, 18]. Others have found few group-based differences, but high individual variability in awareness declines when studied over one year [23], potentially because severity and awareness are mediated by cognitive reserve [24]. Other important clinical correlates of awareness include depression [18, 21, 22, 25], neuropsychiatric status [19–21, 26] caregiver burden [10, 11, 14, 26], activities of daily living [19], and neuropsychological status [12, 19, 20, 22, 27–29] which demonstrate variability in associations with awareness across domains and measurement methods [1].

Models of awareness suggest awareness is mediated by the frontal lobes [2, 3] or the right frontal lobe [30, 31], and lack of awareness is associated with other behavioural indicators of frontal dysfunction, such as increased apathy [32]. Localized imaging has implicated the orbitofrontal cortex [33], but most have implicated medial structures [34, 35], including the anterior [36] and posterior cingulate [35] in awareness. Despite converging evidence for prefrontal involvement in awareness, awareness may or may not be associated with tests of executive function (see review by [1]). Some studies demonstrate strong relationships between awareness and executive functioning [12, 19, 20, 27], with others reporting nonsignificant and trivial associations [19, 29]. Although clearly composite measures that rely on more basic cognitive functions, many summary scores from traditional tests of executive function are associated with the dorsolateral prefrontal circuit [37], and awareness has been associated with more midline [34–36] or orbitofrontal aspects of the prefrontal cortex [33].

Contradictory data on the clinical correlates of awareness is also due to the assumption that awareness is a unitary construct (see reviews by [4, 8]). Differential patterns of clinical correlates have been demonstrated for awareness of cognitive deficits versus awareness of behaviours [12–15], suggesting that awareness is modality specific.

The primary purpose of this paper is to describe the clinical correlates of awareness of balance in addition to the more commonly measured awareness of function in basic and instrumental activities of daily living (BADLs and IADLs, resp.) and awareness of memory. Awareness of balance is important in persons with dementia due to its relation with fall risk [38]. We hypothesize that awareness of balance will be associated with physical variables, such as gait, falls, and objectively measured balance. Based on the conceptualization of Markova and colleagues [39] and work suggesting differential clinical correlates for awareness of specific domains [13, 15], we hypothesize that awareness of functional abilities, memory, and balance will have differential patterns of clinical correlates.

A secondary purpose of this paper is to contrast awareness for balance, function, and memory for those diagnosed with mild cognitive impairment (MCI). Reduced awareness has been demonstrated for persons at high risk for dementia, and specifically for persons with amnestic MCI (aMCI) [40]. Moreover, since awareness appears to differ based on dementia subtype [28], awareness for balance, function, and memory could be differentially affected for participants diagnosed with dementia due to AD versus non-AD dementias. Therefore, the final comparison will explore awareness in groups of persons diagnosed with aMCI, non-amnestic MCI, AD, and non-AD dementias.

## 2. Method

### 2.1. Participants

Patients were from an interdisciplinary memory clinic established to provide early stage dementia differential diagnoses for rural persons [41]. For this IRB approved study, patients who were diagnosed with no cognitive impairment were excluded, and only patients who received a diagnosis of dementia or a variant of MCI were included. Diagnoses in this specialty clinic were consistent with the review of diagnostic guidelines provided by the Canadian Consensus on the Diagnosis and Treatment of Dementias [42] using recent comprehensive blood work, CT head scan, and interprofessional assessment data from neurology, neuropsychology, and physical therapy. The assessment procedures included standardized approaches (e.g., questionnaires, neuropsychological testing) and also family interviews for clinical history and interviews with the informal caregiver who accompanied patients to the clinic (families were strongly encouraged to attend the assessment and patients were asked to bring someone who knew them well. In the unusual circumstance when patients attended the clinic alone, telephone interviews were conducted with someone who knew them well, but questionnaire data were not collected). The sample consisted of 259 patients (in the clinic's 6th data release), and the vast majority (74%) reported their ancestry as European, 9% were First Nations or Metis, and 17% chose “other,” rather than selecting one of the aforementioned categories, African or Asian ancestry.  Table 1 includes descriptive information for the total clinical sample (*N* = 259 patients) and details the subgroups based on diagnosis. Patients with dementia due to AD was the most common diagnosis (*n* = 113), and another heterogeneous subgroup (*n* = 100) was created of patients with non-AD dementias (i.e., vascular dementias, mixed dementias, diffuse Lewy body disease, dementia due to Parkinsons' Disease, Huntington's dementia, variants of frontotemporal lobar degeneration, and dementias not otherwise specified). In addition, a third group included patients diagnosed with amnestic (single or multiple domain) mild cognitive impairment (aMCI; *n* = 23) and a fourth group included patients diagnosed with nonamnestic MCI (non-aMCI; *n* = 23; single or multiple domains, which included those with diagnoses of vascular cognitive impairment, no dementia [43]). Although most of the clinic data were focused on patients for diagnostic purposes, informal caregivers (*N* = 244) provided important collateral and personal information. Caregivers (M age = 61.40, SD = 14.63) were typically family members and most of many were females (64%): 33% were wives, 20% were husbands, 31% were daughters, 10% were sons of the patient, with a remaining 7% whose relationship status included grandchildren, nieces, nephews, or friends.

### 2.2. Measures

*Clinical Correlates of Awareness Measures*

*(1) Assessment of Severity.* The Clinical Dementia Rating (CDR) [44] is a standardized and psychometrically sound clinician-based rating scale (0 to 3; no impairment to severe impairment), but summing the box scores of the six rating areas of the CDR (CDR-SOB) provides a more detailed quantitative measure of global dementia severity and is more sensitive to detecting changes in dementia severity over time [45].

*(2) Assessment of Activities of Daily Living*. Patients rated their performance on the reliable and valid Lawton Instrumental Activities of Daily Living (IADL [46] higher scores indicating independent functioning). Patients' caregivers rated patients' performance of ADLs on two psychometrically strong scales: The Functional Assessment Questionnaire (FAQ) [47] and the Bristol ADL questionnaire [48] where higher scores indicate impaired performance.

*(3) Assessment of Depression and Neuropsychiatric Symptoms*. The Neuropsychiatric Inventory (NPI) is a well-researched and psychometrically strong caregiver rating of patients' behaviours and associated caregiver distress [49]. The Centre for Epidemiologic Studies of Depression (CESD), a reliable and valid screen of depression, was self-rated by patients [50], with four factors: (1) depressed affect (2) lack of positive affect (3) somatic/vegetative and (4) interpersonal measuring social disconnectedness.

*(4) Assessment of Caregiver Psychological Distress and Burden*. Self-report of caregiver burden was assessed with the short form of the Zarit Burden interview, which was shown to be psychometrically similar to the longer versions [51]. The Global Severity Index from the Brief Symptom Inventory (BSI) measured caregiver self-report of overall psychological distress [52].

*(5) Assessment of Physical Variables*. A comprehensivephysical therapy assessment included the psychometrically strong Berg Balance Scale (BBS) [53] and the Performance Oriented Mobility Assessment (POMA, which is a measure of gait and balance) [54]. Caregiver and patient reports of falls within the past 6 months were combined with BBS to estimate the probability of falling [55]. Patient self-report on the Activities-Specific Balance Confidence (ABC) scale [56] measured self-reported confidence in balance while doing a variety of day-to-day activities.

*(6) Assessment of Neuropsychological Function*. Each patient received a comprehensive neuropsychological assessment (see [57] for a review of the strong psychometric properties of these tests), and selected measures of executive function and working memory were analyzed. The ability to alternate attention was measured with the Trail Making Test Part B (TMT B) [58]. The ability to inhibit an automatic response was measured with the Stroop interference score [59]. Cognitive flexibility with speeded retrieval of language-based knowledge was measured using Animal Naming and phonemic fluency (Benton Oral Word Association Test [60]). The clock drawing test measured visual construction, abstraction, and inhibition [61]. Digit span backward subtest from the Wechsler Adult Intelligence Scale 3rd edition (WAIS-III) [62] measured working memory. Finally, the index scores from the Repeatable Battery for the Assessment of Neuropsychological Status (RBANS) [63] were analyzed.

*(7) Awareness of Functional Deficits*. Awareness of functional deficits (AF) was operationalized using patient/caregiver congruence on reports of the patient's ability to independently perform six IADLs: management of finances, use of telephone, use of transportation, shopping, meal preparation, and performance of housework (patient self-report version of the Lawton IADL Scale [46]; and caregiver report of patient's function from the Bristol ADL Scale [48]). For each IADL, congruence was ranked on a 5-point scale; the congruence ranking was summed across the six IADL items for a total of 30 possible points, with higher scores indicating greater awareness. Data were available for 201 participants, and as can be seen in Table 1, most patients had good awareness of their functional abilities. AF was significantly higher in the two MCI diagnostic groups when compared with the two groups with dementia diagnoses.

*(8) Awareness of Memory*. Awareness of memory (AM) was based on congruence between patient's self-reports of memory on a standardized scale (Self-Rating of Memory Scale) [64] and performance on a neuropsychological test of new learning (Repeatable Battery for Assessment of Neuropsychological Status; RBANS delayed memory index score [63]). The delayed memory measure was chosen since it best captured consolidation difficulties asked about in the Self-Rating of Memory Scale. Both the self rating of memory standardized scores and the RBANS index scores were transformed into a linear ranked scale from 1 to 5, with 1 indicating the lowest self-rating of memory and the lowest memory performance. The AM score was created based on the congruence in ranking between self-reported and objectively measured memory, with 5 indicating perfect congruence. Complete data were available for 192 participants. As can be seen in Table 1, most participants' AM was at the mid-point of the scale, but the AD and the aMCI subgroups reported significantly lower AM than the non-AD dementia and non-aMCI groups.

*(9) Awareness of Balance*. Awareness of balance (AB) was based on congruence between patients' ratings of balance confidence on the ABC scale [56] and the probability of falling [55]. The ABC and probability of falling were each transformed into ranked scores with 1 indicating low confidence or high probability of falling and 4 representing high confidence or low probability of falling. The AB score was created based on the congruence in ranking between self-reported balance confidence and objectively measured probability of falling, with 1 indicating low congruence and 4 indicating perfect congruence. Perfect congruence between balance confidence and objective measurement may not be sufficient for stability; rather underestimation of balance (i.e., less confidence than objective measurement would support) has been shown to be associated with greater stability (e.g., [38, 65]). Consistent with this premise, AB ranking of 5 represented an underestimation of balance confidence relative to objectively measured balance. Complete data were available to create AB for 95 participants. Approximately one third of the sample (36%) reported equivalent balance confidence to measured stability, 27% reported greater balance confidence than would be supported by objective measurement (i.e., reduced awareness), and 37% reported an underestimation of balance, which may be appropriate awareness for maximal stability (e.g., [38, 65]). Although the sample size was relatively small, AB was high for all groups and did not differ significantly for patients with diagnoses of aMCI, non-aMCI, AD, or non-AD dementias (see Table 1).

### 2.3. Statistical Procedure

Zero-order correlations were completed separately for AF (Table 2), AM (Table 3), and AB (Table 4). Only variables with significant correlations were used to minimize specification errors (potential over- or undercorrection) as predictors in simultaneous multiple regression equations. Variables with variance inflation factors over 5.0 were excluded due to concerns about multicollinearity. For each measure of awareness, analyses were conducted for the overall sample, but also for each of the four diagnostic groups. Descriptors of magnitude of association were consistent with guidelines for small, medium, and large effect sizes provided by Cohen [66].

## 3. Results

### 3.1. Evidence for Validity of Awareness Measures

As can be seen in Table 2, AF was highly correlated with caregiver report of ADLs (FAQ and Bristol ADL each large magnitude associations). The correlation with patient report of IADL was a moderate magnitude. Together these data suggest AF was highly associated, but not redundant, with more comprehensive measures of day-to-day function.

Similarly, AM was highly associated, but not redundant with the measures used to create it. As can be seen in Table 3, these associations were strong for the non-AD dementia and two MCI groups but trivial for the AD group.

Finally, AB also demonstrated moderate, but nonredundant, associations with the variables used to create it. As can be seen in Table 4, the association with the ABC scale was a moderate magnitude overall and for all diagnostic groups except the non-aMCI group. Also seen in Table 4, AB was associated with both fall history in the past 6 months for the larger samples and overall. AB was associated with probability of falling only for specific diagnostic groups and the overall sample. The cell sizes for the POMA were below 10 for the MCI groups, but the small association was significant for the overall sample.

Overall, the correlations provide evidence for the convergent validity for each of the derived awareness measures. Of interest, each measure of awareness appeared orthogonal: awareness of function was not associated with either awareness of memory (*r*_*s*_ = 0.026, *P* > 0.05, trivial magnitude) or balance (*r*_*s*_ = 0.207, *P* > 0.05, small magnitude), and the latter two measures of awareness are similarly not well associated (*r*_*s*_ = 0.153, *P* > 0.05, small magnitude).

### 3.2. Clinical Correlates for Awareness Measures

#### 3.2.1. Awareness of Function

Zero-order correlations for AF are provided in Table 2. For the overall sample, regression diagnostics suggested the Bristol ADL-caregiver, NPI-Distress, and RBANS immediate and total scale indices were multicollinear, so these variables were excluded from the regression equation. The remaining predictors of CDR-SOB, IADL-Patient, FAQ-caregiver, NPI-severity, ZBI, probability of Falling, BBS, clock drawing, RBANS language, and visuospatial/constructional Indices accounted for a large proportion of AF variance (*R* = 0.688, *P* < 0.001). Not all predictors were equally predictive, however, and only the FAQ-caregiver (*t* = −4.64, *P* < 0.001) and ZBI (*t* = −3.06, *P* = 0.003) were significant predictors of AF. Equivalent regression procedures were conducted separately for the four diagnostic subgroups, and across these analyses only measures of function were significant predictors of AF (AD group FAQ-caregiver *t* = −3.29, *P* = 0.002; non-AD group FAQ-caregiver *t* = −3.03, *P* = 0.004; aMCI group IADL-patient *t* = 2.30, *P* = 0.035; and non-aMCI group BADL-caregiver *t* = −2.37, *P* = 0.029).

#### 3.2.2. Awareness of Memory

Awareness of memory demonstrated a different pattern of zero-order correlations (see Table 3), and the results of the regression analyses also suggested that the clinical correlates of AM clearly differed from those of AF. For the overall sample, the initial regression diagnostics resulted in removal of NPI-severity and distress in addition to the RBANS total scale index score due to multicollinearity. The overall model accounted for a large proportion of variance in AM (*R* = 0.736, *P* < 0.001), but of the predictors (IADL-Patient, CESD overall, CESD depressed affect, CESD somatic/vegetative, CESD interpersonal, BSI global severity, animal naming, and RBANS immediate memory were excluded) only the CESD-Lack of positive affect (*t* = 3.19, *P* = 0.002), the RBANS visuospatial/constructional index (*t* = 2.63, *P* = 0.01), and the RBANS delayed memory index (*t* = 8.65, *P* < 0.001) remained significant. The regression equations for the diagnostic groups differed from the predictors for the overall group. For the non-aMCI group the overall model was nonsignificant (likely due to only 9 participants having all variables complete). For the non-AD dementia group the RBANS delayed memory index was the only significant predictor of AM (*t* = 2.32, *P* = 0.028), but for the few aMCI patients with all predictors complete the delayed memory was not significant (*t* = 2.22, *p* = 0.053). Perhaps most salient was the radically different associations between AM and the clinical correlates for the AD group. Here, only the IADL-Patient (*t* = −2.15, *P* = 0.035) and CESD lack of positive affect (*t* = 2.32, *P* = 0.024) predicted AM.

#### 3.2.3. Awareness of Balance

The zero-order correlations that drove the regression equations for AB are shown in Table 4. The regression equations for the overall sample were less plagued by small sample size problems than the two MCI groups. The clinical correlates of probability of falling, POMA, and the BBS were excluded, however, due to multicollinearity. The remaining predictors were all significant: FAQ-caregiver (*t* = −4.77, *P* < 0.001), the ABC scale (*t* = 6.61, *P* < 0.01), and fall history in Last 6 Months (coded yes/no, *t* = −8.01, *P* < 0.001) and together accounted for a large proportion of variance in AB (*R* = 0.757, *P* < 0.001). When compared across the diagnostic groups, the two regression equations for the MCI groups were not statistically significant, likely due to small sample sizes. For both dementia groups the ABC scale was a significant predictor of AB (AD group *t* = 4.61, *P* = 0.038, non-AD *t* = −2.34, *P* = 0.014) but, in addition, for the non-AD group the fall history in Last 6 Months was significant (*t* = −3.81, *P* = 0.001).

## 4. Discussion

These data support the hypothesis that awareness for different domains, specifically awareness of function, memory, and balance, would differentially relate to clinical correlates. This is in keeping with early work on awareness demonstrating differential clinical correlates for specific awareness domains [12–15] and provides support for the assertion by Markova and colleagues [39] that awareness must be conceptualized as specific to the domain being measured, and that research on one domain cannot be generalized to another domain. In addition to finding differential patterns of clinical correlates across domains of awareness, these data suggest that diagnostic group is also an important consideration in the clinical correlates of awareness. This was most evident in the clinical correlates for the AD group versus the non-AD and aMCI groups for awareness of memory. Here, the relationship between specific symptoms of depression and awareness of memory was only evident for the AD group. In contrast, the clinical correlates for the other groups remain restricted to measures of memory. These data suggest the assertion by Markova et al. [39] regarding caution in cross-domain generalization is not sufficient, and diagnostic grouping is another important consideration, at least for some domains of awareness.

Moreover, awareness for specific domains was shown to vary across the diagnostic groups. Awareness of function was lower for the groups diagnosed with dementia than those with MCI whereas awareness of memory was lower for the group with AD dementia and the group with aMCI, often considered a precursor to AD [67], than for the non-AD dementia or non-aMCI groups. Awareness of balance did not appear to differ across the diagnostic subgroups.

Our data suggest that awareness of specific domains was orthogonal: awareness of function was not associated with awareness of memory or awareness of balance. This was in contrast to the findings by Ott and colleagues [12] who found moderate correlations between awareness of memory and awareness of function. Our method for measuring awareness of function was similar to that used by Ott and colleagues (namely, patient/caregiver discrepancy), but we used a discrepancy between performance and self-report to assess memory and balance awareness, which may account for these inconsistent findings.

Evidence for the modality specific nature of awareness is provided by the differential patterns of clinical correlates depending on domain measured. We found that the relation between caregiver burden and distress depended on the modality of awareness measured: reduced awareness of function was associated with increased caregiver reports of burden, which is consistent with findings from previous research [10, 11, 14]. Balance awareness was the only awareness measure associated with physical variables such as past history of falls and self-reported balance confidence. The relation between balance awareness and falls is consistent with previous research demonstrating a strong relationship between proprioception and balance [65] or falls [38]. Differential patterns of awareness were also shown for relations with neuropsychological variables. Balance awareness was not related to any measure of neuropsychological functioning, despite previously reported relationships between risk of falls and measures of executive function [68]. Similarly, awareness of function was not associated with neuropsychological variables. Although none of the domains of awareness were associated with measures of executive function, awareness of memory was associated with neuropsychological variables of the delayed memory index and the visuospatial/constructional indices from the RBANS. The associations with neuropsychological variables differed, however, when the diagnostic groups were considered separately. Interestingly, awareness of memory was not associated with the delayed memory scores for the AD group. A floor effect in the delayed memory score appears to have created problems with heteroscedasticity in the bivariate memory awareness relationship, which may have attenuated any associations. The possible floor effect in memory measures did not, however, impact the association between depressive symptoms and awareness of memory, only for the AD group.

The findings of differential clinical correlates for awareness of memory based on diagnostic group may speak to some of the most contradictory findings regarding correlates of awareness. Neuropsychological function is inconsistently related to awareness [1, 12, 19, 20, 27, 29], and the relationship between awareness in dementia and depression is complicated, with clinical lore and empirical data supporting the notion that increased awareness is associated with more symptoms of depressed mood [18, 25], but increased awareness and depression may only be related to subclinical (or dysthymia) rather than major depression [1]. Our data suggest that in addition to modality of awareness being considered when measuring associations of neuropsychological and depressive symptoms with awareness, diagnostic group is an additional important consideration. Patients with dementia due to AD appear to have differential clinical correlates for awareness of memory versus patients with non-AD dementia, for example.

Although the prediction of differential clinical correlates for the different domains of awareness was supported, some of our predictions regarding these clinical correlates were contrary to previous research. In zero-order associations, severity was associated with awareness of function, but with no other domain of awareness. Moreover, severity did not account for sufficient unique variance in awareness of function and was, therefore, not a significant predictor. This finding is in contrast to the cumulative cross-sectional data demonstrating an association between severity of cognitive impairment and awareness (see [1] for a thorough review) and is contrary to the more compelling longitudinal data demonstrating decreasing awareness with increasing cognitive impairment [16, 18]. These contrasting findings likely speak to the orthogonal and domain-specific nature of the construct of awareness. Aalten and colleagues' [18] and McDaniel and colleagues' [16] prospective studies measured awareness by clinician ratings based on an interview with patient and caregiver regarding the patient's history of cognitive deficits and their impact on function.

Finally, all measures of awareness were associated with some measure of independence in daily function, particularly instrumental activities of daily living which is evidence against modality specificity. Assessment of functional abilities as a clinical correlate for measures of awareness is not often reported in the literature, but the few studies that do exist suggest that decreased awareness is associated with increased functional limitations [19], which is consistent with our data.

Despite adding to the converging research on the modality-specific nature of awareness [4, 8–10], these data are limited by the inconsistency in the measurement methods used for each modality of awareness. Measurement of awareness of function was based on caregiver/patient discrepancy, whereas awareness of memory and of balance was based on discrepancy between self-report and objective measures of performance. If awareness is a true construct, it should not be highly dependent on how it is measured. If, for example, awareness of function differs greatly when measured with caregiver/patient discrepancy versus patient/observation discrepancy, then this would not be a construct at all and would be considered an artifact of measurement [69]. If awareness is an artifact of measurement, future research measuring awareness of different modalities of awareness with different methods for measuring awareness will find markedly different clinical correlates than those presented here.

Another more problematic limitation of these data is the fact that this sample is derived from a specialty clinic. Specialty clinic patients have been postulated to have higher awareness than the general dementia population due to the referral process for specialty clinics [70]. It is unclear how having higher awareness may have impacted the differential patterns of clinical correlates for these multiple domains of awareness.

Although replication is required, these data demonstrate differential patterns of clinical correlates for awareness of function and memory. In addition, this study provides a novel contribution by describing the clinical correlates for awareness of balance. These data provide evidence for modality specific relations between awareness and clinical correlates in a database with a wide range of standardized measures of clinical correlates including severity, function, neuropsychiatric symptoms, depression, caregiver burden and distress, and a comprehensive assessment of neuropsychological status. Differing patterns of clinical correlates for awareness of function, awareness of memory, and awareness of balance provide support for the modality specific uniqueness of awareness measures for each of these domains. Data demonstrating different patterns of awareness based on diagnostic group (namely, aMCI, non-aMCI, AD, and non-AD dementia diagnoses) provides further evidence for the modality specific nature of awareness. Modality specificity, if replicated in different populations, for example, stroke patients, could have implications for rehabilitation. These data would suggest that rehabilitation needs to be targeted to a domain of awareness since awareness is not a unitary construct. Future research is needed on the clinical course and implications for day-to-day care associated with impairments in specific modalities of awareness for persons with dementia.

## Figures and Tables

**Table 1:** Descriptive statistics for demographic, awareness, and clinical variables for each of the four diagnostic subgroups and for the overall sample.

	AD M (SD) *n *	Non-AD M (SD) *n *	aMCI M (SD) *n *	Non-aMCI M (SD) *n *	Overall M (SD) *n *
Age	75.81(7.51) 113	73.05 (10.15) 100	73.74 (8.87) 23	70.30 (11.06) 23	74.07 (9.18)^a^ 259
Gender (% female)	67%	56%	70%	52%	62%
Education	10.16 (3.32) 108	10.57 (3.168) 87	11.13 (3.57) 23	11.05 (3.43) 23	10.48 (3.30) 240
Awareness of function	24.74 (3.53) 87	23.75 (3.91) 72	26.30 (3.06) 20	27.59 (3.02) 22	24.85 (3.76)^b^ 201
Awareness of memory	2.13 (0.99) 88	2.69 (1.20) 61	2.55 (1.10) 22	3.10 (1.18) 21	2.46 (1.14)^c^ 192
Awareness of balance	4.07 (0.99) 43	3.81 (0.92) 27	4.00 (0.91) 13	4.42 (0.79) 12	4.03 (0.94) 95
CDR-SOB	6.97 (3.45) 100	6.45 (3.58) 91	2.13 (1.310) 20	2.27 (1.22) 22	5.90 (3.66)^b^ 233
IADL-patient	21.64 (5.05) 99	20.11 (5.47) 82	24.68 (2.71) 22	25.32 (2.73) 22	21.74 (5.14)^b^ 225
FAQ-caregiver	15.52 (8.11) 109	15.36 (8.29) 94	7.82 (4.81) 22	6.65 (6.33) 23	13.96 (8.38)^b^ 248
Bristol ADL-caregiver	8.90 (7.50) 102	10.97 (9.78) 89	2.96 (2.06) 23	4.04 (5.30) 23	8.63 (8.37)^b^ 237
CESD overall	11.85 (9.46) 93	13.31 (9.10) 72	15.00 (8.54) 23	14.05 (7.86) 22	12.92 (9.08) 210
CESD depressed affect	2.91 (3.30) 93	2.89 (3.21) 72	3.70 (3.43) 23	2.68 (2.38) 22	2.97 (3.19) 210
CESD Lack of positive affect	2.75 (2.86) 93	3.35 (2.74) 72	4.00 (3.44) 23	3.41 (2.61) 22	3.16 (2.87) 210
CESD somatic/vegetative	5.08 (3.92) 93	5.96 (3.97) 72	5.96 (3.78) 23	6.73 (4.20) 22	5.65 (3.96) 210
CESD interpersonal	1.11 (1.77) 93	1.11 (1.44) 72	1.35 (1.90) 23	1.23 (1.66) 22	1.15 (1.65) 210
NPI-severity	8.70 (6.49) 103	9.24 (5.81) 84	15.50 (41.27)* 20	7.44 (8.03) 16	9.42 (13.64) 223
NPI-distress	10.08 (10.02) 103	10.57 (8.74) 82	14.45 (39.23)* 22	7.00 (11.96) 20	10.41 (15.18) 227
ZBI	13.76 (8.97) 107	14.25 (8.35) 89	11.73 (9.35) 22	10.35 (9.56) 23	13.43 (8.86) 241
BSI global severity	50.35 (10.05) 99	52.03 (9.30) 86	51.24 (10.51) 21	53.22 (11.34) 23	51.35 (9.93) 229
Fall history in past 6 months (no/yes)	65/25 90	45/36 81	12/8 20	10/12 22	132/81 213
Probability of falling (%)	38.00 (35.06) 76	56.02 (33.87) 63	32.84 (32.48) 17	39.45 (37.13) 14	44.28 (35.44) 170
ABC	80.95 (16.99) 44	74.40 (24.87) 29	86.15 (11.09) 13	72.92 (16.46) 12	78.72 (19.28) 98
BBS	46.44 (8.57) 77	41.97 (11.48) 64	49.53 (5.03) 17	49.14 (7.23) 14	45.30 (9.76)^d^ 172
POMA	22.25 (4.85) 32	20.38 (5.54) 26	21.88 (4.70) 8	23.50 (4.09) 6	21.64 (5.05) 72
Stroop-interference	−0.90 (1.55) 79	−1.19 (1.64) 54	−0.43 (1.44) 21	0.21 (0.86) 18	−0.82 (1.55)^e^ 172
TMT-B	−2.10 (1.26) 62	−2.32 (1.15) 48	−0.92 (1.00) 20	−1.68 (1.43) 17	−1.96 (1.29)^f^ 147
Clock drawing	16.30 (4.28) 100	15.85 (4.79) 79	18.98 (1.86) 23	17.71 (4.43) 21	16.55 (4.39)^b^ 223
Phonemic fluency	−1.38 (1.00) 98	−1.93 (1.05) 75	−0.81 (1.05) 23	−1.52 (0.98) 22	−1.52 (1.07)^d^ 218
Animal naming	−1.99 (0.78) 98	−2.01 (0.84) 77	−1.27 (1.02) 23	−1.26 (1.03) 22	−1.85 (0.90)^b^ 220
DS-B	−1.04 (0.87) 98	−1.14 (0.82) 76	−0.31 (1.00) 23	−0.82 (0.81) 22	−0.98 (0.89)^b^ 219
RBANS immed memory	57.47 (13.65) 98	65.59 (16.66) 73	75.48 (11.95) 23	82.95 (15.04) 22	64.73 (16.87)^g^ 216
RBANS delayed memory	48.68 (9.35) 95	63.01 (19.13) 70	61.77 (16.89) 22	73.24 (22.74) 21	57.37 (17.71)^h^ 208
RBANS language	78.00 (14.81) 98	78.96 (15.72) 74	94.30 (10.07) 23	94.29 (7.98) 21	81.65 (15.51)^b^ 216
RBANS attention	74.98 (16.39) 91	70.20 (14.85) 64	86.68 (10.79) 22	77.15 (15.58) 20	74.95 (15.92)^i^ 197
RBANS visuospatial/constructional	77.37 (17.60) 93	76.81 (15.83) 67	96.57 (14.83) 21	86.71 (12.79) 21	80.15 (17.38)^i^ 202
RBANS total scale	61.16 (10.09) 83	66.03 (13.73) 59	77.41 (7.67) 22	78.79 (12.26) 19	66.51 (13.07)^b^ 183

^a^AD group significantly older than non-aMCI group.^ b^AD and non-AD dementia group similar, but significantly more impaired than the similar aMCI and non-MCI groups. ^c^AD group similar to aMCI group, but more impaired than non-AD and non-aMCI groups. ^d^Non-AD group more impaired than other groups.^ e^non-aMCI group less impaired.^ f^aMCI group less impaired. ^g^AD group significantly more impaired than non-AD group and both more impaired than either MCI group. ^h^AD group significantly more impaired than other groups. ^i^AD group significantly lower than aMCI group. *outliers in these small samples.

**Table 2:** Clinical correlates of awareness of function (AF) for each diagnostic group.

	AD *r*_*s*_, *n *	Non-AD *r*_*s*_, *n *	aMCI *r*_*s*_, *n *	Non-aMCI *r*_*s*_, *n *	Overall *r*_*s*_, *n *
CDR-SOB	−0.443, 78**	−0.292, 69*	−0.177, 17	−0.121, 21	−0.435, 185**
IADL-patient	0.274, 87*	0.276, 72*	0.688, 20**	0.575, 22**	0.406, 201**
FAQ-caregiver	−0.543, 87**	−0.454, 72**	−0.517, 20*	−0.624, 22**	−0.583, 201**
Bristol ADL-caregiver	−0.651, 87**	−0.513, 72**	−0.651, 20**	−0.632, 22**	−0.655, 201**
CESD overall	−0.005, 79	0.012, 62	0.218, 20	0.338, 21	0.076, 182
CESD depressed affect	−0.023, 79	−0.081, 62	0.303, 20	−0.032, 21	0.007, 182
CESD lack pos affect	−0.071, 79	0.191, 62	0.120, 20	0.496, 21*	0.092, 182
CESD somatic/vegetative	0.064, 79	−0.109, 62	0.335, 20	0.231, 21	0.043, 182
CESD interpersonal	0.070, 79	0.113, 62	0.104, 20	0.127, 21	0.088, 182
NPI-severity	−0.202, 83	−0.073, 64	−0.210, 17	−0.511, 15	−0.236, 179**
NPI-distress	−0.276, 83*	0.020, 61	−0.106, 19	−0.436, 19	−0.270, 182**
ZBI	−0.321, 86**	−0.055, 70	−0.437, 20	−0.453, 22*	−0.305, 198**
BSI global severity	−0.037, 78	0.022, 69	−0.087, 19	−0.003, 22	−0.003, 188
Fall history in past 6 months	−0.011, 73	−0.146, 62	0.144, 18	−0.668, 21**	−0.078, 174
Probability of falling (%)	−0.012, 62	−0.160, 48	0.036, 15	−0.815, 14**	−0.188, 139*
ABC	−0.046, 37	−0.127, 24	0.232, 12	0.538, 12	−0.025, 85
BBS	0.028, 63	0.136, 49	0.263, 15	0.640, 14*	0.213, 141*
POMA	0.277, 25	0.177, 22	—	—	0.253, 59
Stroop-interference	−0.006, 65	−0.060, 45	−0.164, 18	0.257, 17	0.147, 145
TMT-B	0.086, 51	−0.020, 43	−0.074, 17	0.015, 16	0.135, 127
Clock drawing	0.188, 81	0.172, 63	0.166, 20	0.350, 20	0.304, 184**
Phonemic fluency	0.099, 79	0.080, 61	−0.102, 20	0.075, 21	0.128, 181
Animal naming	0.022, 79	0.017, 63	−0.114, 20	0.205, 21	0.128, 183
DS-B	0.092, 81	0.037, 62	0.043, 20	−0.357, 21	0.071, 184
RBANS immed memory	0.063, 80	0.091, 60	0.130, 20	−0.096, 21	0.164, 181*
RBANS delayed memory	0.182, 77	0.065, 57	−0.360, 19	0.147, 20	0.125, 173
RBANS language	0.244, 80*	0.106, 60	−0.047, 20	0.365, 20	0.287, 180**
RBANS attention	0.006, 74	0.196, 51	−0.012, 19	0.020, 19	0.104, 163
RBANS visuospatial/constructional	0.110, 75	0.166, 53	0.328, 19	−0.184, 20	0.205, 167**
RBANS total scale	0.137, 66	0.202, 48	0.023, 19	0.039, 18	0.246, 151**

*P < 0.05 but > 0.01. **P < 0.01.

**Table 3:** Clinical correlates of awareness of memory (AM) for each diagnostic group.

	AD *r*_*s*_, *n *	Non-AD *r*_*s*_, *n *	aMCI *r*_*s*_, *n *	Non-aMCI *r*_*s*_, *n *	Overall *r*_*s*_, *n *
CDR-SOB	0.034, 83	0.067, 59	0.208, 19	−0.218, 20	−0.058, 181
IADL-patient	−0.293, 85**	−0.062, 60	−0.221, 22	−0.253, 20	−0.163, 187*
FAQ-caregiver	0.185, 88	−0.053, 61	0.011, 21	0.264, 21	0.009, 191
Bristol ADL-caregiver	0.079, 82	0.087, 57	0.022, 22	0.120, 21	0.032, 182
CESD overall	0.537, 83**	0.226, 58	−0.023, 22	0.071, 20	0.333, 183**
CESD depressed affect	0.326, 83**	0.021, 58	−0.085, 22	0.188, 20	0.172, 183*
CESD lack of positive affect	0.522, 83**	0.179, 58	−0.054, 22	−0.061, 20	0.276, 183**
CESD somatic/vegetative	0.450, 83**	0.265, 58*	−0.094, 22	0.191, 20	0.330, 183**
CESD interpersonal	0.343, 83**	0.218, 58	0.249, 22	0.120, 20	0.272, 183**
NPI-severity	0.163, 84	0.108, 55	0.457, 19*	0.460, 15	0.211, 173**
NPI-distress	0.201, 88	0.123, 59	0.443, 21*	0.018, 21	0.171, 189*
ZBI	−0.002, 88	0.242, 59	0.149, 21	0.147, 21	0.092, 189
BSI global severity	0.278, 81*	0.517, 58**	−0.135, 20	0.042, 21	0.303, 180**
Fall history in past 6 months	0.059, 68	0.039, 46	−0.114, 19	−0.173, 20	0.080, 153
Probability of falling (%)	0.020, 58	−0.154, 36	−0.045, 16	−0.568, 12	−0.010, 122
ABC	−0.112, 34	0.134, 21	−0.555, 13*	0.182, 11	−0.097, 79
BBS	−0.010, 58	0.213, 37	0.001, 16	0.723, 12**	0.090, 123
POMA	0.064, 22	0.309, 16	—	—	0.229, 51
Stroop-interference	−0.081, 71	0.175, 44	0.298, 20	−0.177, 17	0.112, 152
TMT-B	−0.129, 55	0.428, 43**	−0.040, 19	−0.087, 16	0.111, 133
Clock drawing	−0.123, 86	0.202, 58	0.118, 22	0.270, 20	0.078, 186
Phonemic fluency	−0.065, 84	0.153, 59	0.159, 22	0.135, 21	0.010, 186
Animal naming	0.025, 83	0.306, 60*	0.182, 22	0.523, 21*	0.209, 186**
DS-B	−0.133, 87	0.023, 60	0.239, 22	0.087, 21	0.009, 190
RBANS immed memory	−0.089, 88	0.294, 61*	0.273, 22	0.737, 21**	0.232, 192**
RBANS delayed memory	0.005, 85	0.547, 58**	0.450, 21*	0.710, 20**	0.381, 184**
RBANS language	−0.102, 87	0.192, 60	0.065, 22	0.248, 20	0.107, 189
RBANS attention	−0.182, 79	0.347, 52*	0.038, 21	0.217, 19	0.053, 171
RBANS visuospatial/constructional	0.130, 81	0.397, 54**	0.023, 20	0.328, 20	0.252, 175**
RBANS total scale	−0.079, 74	0.527, 49**	0.359, 21	0.768, 18**	0.334, 162**

*P < 0.05 but > 0.01. **P < 0.01.

**Table 4:** Clinical correlates of awareness of balance (AB) for each diagnostic group.

	AD *r*_*s*_, *n *	Non-AD *r*_*s*_, *n *	aMCI *r*_*s*_, *n *	Non-aMCI *r*_*s*_, *n *	Overall *r*_*s*_, *n *
CDR-SOB	−0.001, 37	−0.024, 24	−0.606, 10	−0.543, 11	−0.176, 82
IADL-patient	0.268, 39	−0.222, 24	−0.464, 13	0.063, 12	0.013, 88
FAQ-caregiver	−0.261, 41	−0.142, 26	−0.027, 13	−0.614, 12*	−0.253, 92*
Bristol aDL-caregiver	0.003, 41	−0.036, 26	−0.064, 13	−0.440, 12	−0.138, 92
CESD overall	−0.021, 38	0.345, 22	0.268, 13	−0.099, 12	0.109, 85
CESD depressed affect	−0.022, 38	0.063, 22	0.403, 13	−0.182, 12	0.070, 85
CESD lack of positive affect	0.006, 38	0.339, 22	−0.295, 13	0.090, 12	0.069, 85
CESD somatic/vegetative	0.051, 38	0.316, 22	0.182, 13	0.159, 12	0.139, 85
CESD interpersonal	−0.079, 38	0.296, 22	0.061, 13	−0.064, 12	0.028, 85
NPI-severity	−0.114, 39	−0.083, 26	0.149, 12	—	−0.064, 86
NPI-distress	−0.061, 38	−0.017, 25	0.244, 12	−0.400, 10	−0.073, 85
ZBI	−0.238, 41	0.171, 25	0.074, 13	−0.416, 12	−0.196, 91
BSI global severity	−0.033, 37	0.271, 23	−0.292, 12	−0.168, 12	−0.002, 84
Fall history in past 6 months	−0.570, 43**	−0.569, 27**	−0.331, 13	−0.544, 12	−0.512, 95**
Probability of falling (%)	−0.549, 43**	−0.372, 27	−0.428, 13	−0.612, 12*	−0.501, 95**
ABC	−0.368, 43*	−0.480, 27*	−0.554, 13*	−0.109, 12	−0.392, 95**
BBS	0.310, 43*	0.049, 27	0.326, 13	0.593, 12*	0.289, 95**
POMA	0.324, 24	0.101, 17	—	—	0.316, 54*
Stroop-interference	−0.201, 35	0.203, 22	0.164, 12	−0.152, 12	0.076, 81
TMT-B	0.118, 19	0.051, 11	0.294, 10	−0.116, 10	0.063, 50
Clock drawing	−0.129, 39	0.077, 25	0.029, 13	0.144, 12	0.006, 89
Phonemic fluency	−0.126, 38	−0.033, 25	0.247, 13	0.257, 12	0.009, 88
Animal naming	−0.157, 39	0.133, 25	−0.035, 13	0.680, 12*	0.020, 89
DS-B	0.073, 37	−0.077, 24	0.077, 13	−0.030, 12	0.044, 86
RBANS immed memory	−0.109, 37	−0.091, 24	−0.027, 13	0.271, 12	0.016, 86
RBANS delayed memory	−0.130, 36	−0.056, 21	0.077, 12	0.723, 12**	−0.001, 81
RBANS language	−0.277, 37	0.113, 23	0.362, 13	0.129, 12	0.035, 85
RBANS attention	0.133, 37	0.125, 20	−0.342, 12	0.228, 12	0.072, 81
RBANS visuospatial/constructional	0.047, 37	−0.366, 20	−0.101, 11	0.050, 12	0.012, 80
RBANS Total Scale	−0.022, 34	0.052, 20	0.067, 12	0.583, 11	0.114, 77

*P < 0.05 but > 0.01. **P < 0.01.
